# Quantitation of 5-methyltetraydrofolic acid in plasma for determination of folate status and clinical studies by stable isotope dilution assays

**DOI:** 10.1371/journal.pone.0212255

**Published:** 2019-02-21

**Authors:** Lisa Striegel, Beate Brandl, Markus Kopp, Lukas Sam, Thomas Skurk, Michael Rychlik

**Affiliations:** 1 Chair of Analytical Food Chemistry, Technical University of Munich, Freising, Germany; 2 ZIEL, Institute for Food & Health, Core Facility Human Studies, Technical University of Munich, Freising, Germany; 3 Else Kröner-Fresenius-Center of Nutritional Medicine, Technical University of Munich, Freising, Germany; 4 Institute of Nutritional Medicine, Technical University of Munich, Klinikum rechts der Isar, Munich, Germany; 5 University of Queensland, Centre for Nutrition and Food Sciences, Brisbane, Australia; University of Illinois, UNITED STATES

## Abstract

Folates play a key role in the prevention of neural tube defects in newborns. Thus, it is important to reliably determine the bioavailability of folates from various foods. Accurate analytical methods are essential for quantifying blood-folates, especially in human studies. Here, we present the development and validation of a sensitive method using stable isotope dilution liquid chromatography coupled with mass spectrometry for determining various folates in plasma. Moreover, this study reports the applicability of the developed method to a human pilot study using strawberries as a test food. Validation of the assay revealed the precision, sensitivity, and accuracy of the method in determining the predominant 5-methyltetrahydrofolate in plasma. This method was also applicable for the screening of individual folate status using finger prick blood and for monitoring the post-absorptive plasma-concentration curve. Moreover, the human study revealed a high recovery of strawberry folates with a calculated relative bioavailability of 96.2%. Thus, the developed method enables prospective bioavailability studies. This work also confirmed, via human studies, that strawberries are a rich and natural source of folates that are available for human metabolism.

## Introduction

Folates, mainly in the form of polyglutamates, play a key role in one-carbon metabolism, which involves nucleotide biosynthesis, amino acid and DNA synthesis as well as the remethylation of homocysteine to methionine [1]. An increased plasma homocysteine concentration is a known risk factor for cardiovascular diseases [2, 3]. Further, a deficiency of folate post conception and during early pregnancy may cause neural tube defects in newborns [4]. An inadequate folate status might be associated with colorectal cancer [5] and Alzheimer's disease [6]. In societies, particularly in those without mandatory folic acid fortification, such as Germany and several other European countries, the bioavailability of folate from natural sources is an important health concern. The bioavailability of food folate is estimated at 50% [7]. However, human studies have indicated a great variation in the bioavailability of food folate ranging from 30% to 98% compared with chemical-based folic acid products [8, 9]. The bioavailability of food folates is dependent on the food matrix, the degree of folate release from the matrix and also on the presence of other food constituents such as ascorbic acid [10] and folate-binding proteins from milk [11]. The variable outcome of previous bioavailability studies may be attributed to different treatments of the test food mainly since processes such as boiling, leaching, and oven baking may reduce the food folate content [12–18]. Therefore, the total folate content of unprocessed foods is not necessarily that which may finally be available for intestinal absorption. Furthermore, the intestinal hydrolysis of polyglutamates and individual genetic diversity such as single nucleotide polymorphisms of methylenetetrahydrofolate reductase, such as the C677T variation [19], need to be taken into account when assessing the bioavailability of folates. Unfortunately, the factors that influence this bioavailability are poorly understood. Individual foods need to be tested in order to better estimate the bioavailability of food folates after a short-term intake, and more data on the bioavailability of natural folates are needed for dietary recommendations.

The methodological differences for quantification of folate in blood samples to monitor the individual folate status and to accurately detect the time-concentration curve is an important issue. In recent years, high performance liquid chromatography (HPLC) methods linked with mass spectrometry and the implementation of a stable isotope dilution assay (SIDA) have advanced greatly [20–24]. SIDA is based on the addition of isotopically labeled internal standards, which enable a complete compensation for the losses of analytes occurring during clean-up as well as for ion suppression during liquid chromatography coupled with mass spectrometry (LC-MS/MS) measurements [25]. However, more efforts are needed in order to standardize methods for achieving higher comparability.

Strawberries are popular and worldwide consumed as well as important sources of nutritive compounds such as vitamin C, minerals, fibers, and polyphenolic phytochemicals and are consumed worldwide [26, 27]. Our recent report [28] indicated strawberries as a very good potential source of natural folates. The folate content in strawberries was estimated to range from 93 to 153 μg/100 g of fresh weight and, therefore, belong to one of the most abundant folate sources among fruits. In order to evaluate the nutritional value of strawberries, it is necessary to obtain detailed information about the inaccessibility and bioavailability of folate for physiological functions through human studies.

On this backdrop, the aim of our study was to validate a sensitive method for the analysis of plasma folate using the advantageous methodology of LC-MS/MS and SIDA. As an application test, a human short-term study to better understand the availability of folates from strawberry for physiological functions was conducted.

## Materials and methods

### Chemicals

Acetonitrile, methanol, water (LC-MS/MS grade and HPLC grade) were acquired from VWR (Ismaning, Germany). Ascorbic acid, formic acid (>95%), and 2-(N-morpholino)-ethanesulfonic acid (MES) were purchased from Sigma-Aldrich (Steinheim, Germany). Potassium dihydrogen phosphate, sodium acetate trihydrate, and sodium hydroxide were obtained from Merck (Darmstadt, Germany). Disodium hydrogen phosphate (anhydrous) and sodium chloride were obtained from Alfa Aesar and Baker J.T. (Thermo Fisher, Karlsruhe, Germany). Dithiothreitol (DTT) was purchased from Applichem (Darmstadt, Germany). Unlabeled (5-methyltetrahydrofolate, 5-CH_3_-H_4_folate) reference compound was obtained from Schircks Laboratories (Jona, Switzerland), labeled reference compound ([^13^C_5_]-5-CH_3_-H_4_folate) was obtained from Merck & Cie (Schaffhausen, Switzerland); folic acid was purchased from Fluka (Sigma Aldrich, Steinheim, Germany). Strata SAX cartridges (quaternary amine, 100 mg, 1 mL) were obtained from Phenomenex (Aschaffenburg, Germany).

### Buffers and solutions

#### Solutions for extraction

The extraction solution consisted of ascorbic acid (114 mM, MES (200 mM) and DTT (0.7 mM), pH = 5 was adjusted using NaOH. Phosphate buffer of 100 mM was prepared using disodium hydrogen phosphate aqueous solution (100 mM, A) and potassium dihydrogen phosphate (100 mM, B). pH of solution A was adjusted to 7.0 with solution B.

#### Solution for equilibrating (solid phase extraction (SPE) clean-up)

This solution was prepared by mixing phosphate buffer (10 mM) with DTT (1.3 mM).

#### Eluting solution (SPE clean-up)

A buffer consisting of sodium chloride (5%), sodium acetate (100 mM), DTT (0.7 mM), and ascorbic acid (1%) was used as eluting solution.

#### Stock solutions and internal standards

Unlabeled analytes, namely, 5-CH_3_-H_4_folate and pteroylmonoglutamate (PteGlu), were used. PteGlu was used as an internal standard to determine the concentration of 5-CH_3_-H_4_folate by HPLC/DAD. Stock solutions of unlabeled analytes were freshly made before each sample preparation. Using a known concentration of 5-CH_3_-H_4_folate, the concentration of [^13^C_5_]-5-CH_3_-H_4_folate could be assessed by LC-MS/MS. PteGlu (10 mg/100 mL) and 5-CH_3_-H_4_folate (2 mg/10 mL) were dissolved in 10 mL (PteGlu) and 3 mL (5-CH_3_-H_4_folate) of phosphate before adjusting volume with the buffer for extraction. The purity of unlabeled 5-CH_3_-H_4_folate was established using HPLC/DAD and PteGlu as an internal standard. A 1:20 dilution of 5-CH_3_-H_4_folate stock solution was used for LC-MS/MS calibration. The isotope-labeled internal standard [^13^C_5_]-5-CH_3_-H_4_folate was dissolved at a concentration of 200 μmol/L in the extraction buffer. For sample extraction, the internal standard was further diluted to a concentration of 200 nmol/L to fit within the calibration range. The internal standard solution was stored at −20°C in the dark. The stability of the internal standard stock solution was monitored during each extraction. To this end, a mixture of 5-CH_3_-H_4_folate and [^13^C_5_]-5-CH_3_-H_4_folate was freshly prepared and the concentration of the internal standard was established during each extraction by LC-MS/MS.

### Pilot study and ethical permission

#### Ethics statement

Ethical permission was reviewed and approved by the ethics committee of the Faculty of Medicine of the Technical University of Munich (#69/16S). The study was registered in the German Clinical Trial Register (DRKS00010137). Written informed consent was obtained from participants before inclusion into the study.

#### Study participants

Participants with a body mass index (BMI) <30 kg/m^2^ were recruited on a voluntary basis in 2016 and 2017 at the campus of the Technical University of Munich in Freising, Germany. The eligibility of the participants was assessed with a screening questionnaire. Exclusion criteria were BMI >30 kg/m^2^, smokers, acute infections, pregnant or breastfeeding mothers, severe diseases (e.g., cancer), intestinal diseases, allergies or intolerances to the tested food, and carrier of type TT 5-, 10-methylenetetrahydrofolate reductase enzyme.

#### Study design

**Fig 1:**
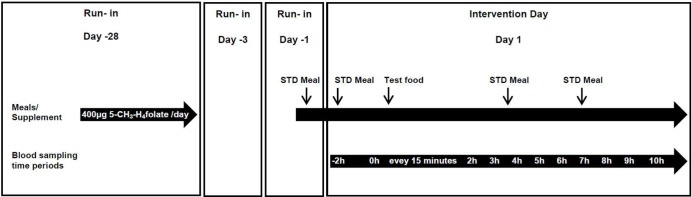
Study design. Overview of the timeline and the different blood sampling time points for one of the three interventions. Details are described in the text. 5-CH_3_-H_4_folate, 5-methyltetrahydrofolate; STD, standardized.

Fig 1 summarizes the study design of the intervention study. Two healthy, non-smoker subjects, female (30 years) and male (25 years), participated in the pilot study. In order to improve uniformity and ensure saturation of storage folates among the two subjects, a 5-CH_3_-H_4_folate supplement (400 μg/day) was administered for at least 4 weeks before the first testing and discontinued 2 days prior to the start of the study day. Between the study days, the subjects took a 5-CH_3_-H_4_folate supplement (400 μg/day) for at least 2 weeks. The study had a randomized cross-over design, and each subject underwent three independent treatments separated by an equilibrium phase of at least 14 days: folate-free test day (600 g of a water-pectin-sugar mixture), strawberry test day (about 600 g blended strawberries), and a folate supplement test day (400 μg 5-CH_3_-H_4_folate supplement served with 600 g of a water-pectin-sugar mixture). Both subjects were required to follow a fixed diet plan. The dinner before each test day consisted of folate-deficient food, namely, chicken legs with polished rice. To avoid transition into a fasting state, the test subjects were required to consume folate-deficient food, namely, rice waffles with honey or Edam cheese, 2 h before the test food was administered and also in regular intervals during the experimental treatment. The consumption of water was allowed ad libitum. Venous blood samples (9 mL, 60 μL were used for extraction) were drawn in K_3_EDTA-coated tubes (Sarstedt, Nürnbrecht, Germany) at defined time intervals: before the administration of the test food, every 15 min for 2 h after the administration of the test food, every hour up to 10 h after the initial 2 h. Blood was immediately centrifuged (10 min, 2.500 rpm, 4°C), and plasma was stored with a saturated amount of ascorbic acid at −20°C in the dark until extraction.

### Sampling procedure

Blood samples (60 μL) were drawn from two healthy non-smoking volunteers. Venous blood was collected in K_3_EDTA Microvettes (Sarstedt, Nürnbrecht, Germany), and plasma was obtained by centrifuging the blood for 10 min at 2500 rpm and 4°C. The plasma was stored in the dark until extraction at −20°C in 1 mL aliquots saturated with ascorbic acid. Blood sampling (30 μL) was performed by finger prick using a single-use lancet (Roche Diagnostics, Mannheim, Germany). Blood drops were collected in Microvette tubes (100 μL, Lithium Heparin coated, Sarstedt, Nürnbrecht, Germany) to avoid clotting. After subsequent plasma centrifugation (10 min, 2.500 rcf, 4°C), plasma aliquots (30 μL) were directly extracted.

### Sample preparation

Plasma samples (60 μL plasma from venous blood and 30 μL plasma from finger prick blood) were added to 1 mL of buffer for extraction. Subsequently, the mixture was spiked with [^13^C_5_]-5-CH_3_-H_4_folate (4 μmol) and equilibrated for 30 min at room temperature in the dark and under constant stirring. A SPE using a strong anion-exchanger (SAX, quaternary amine, 100 mg, 1 mL) was performed for purification. The cartridges were activated with two volumes of methanol and two volumes of equilibrating solution (pH 7). The extracts were applied to cartridges and washed again with two volumes of buffer for equilibration and allowed to run dry. Folates were eluted by using 0.5 mL eluting solution. The final eluate was membrane filtered (PVDF, 0.22 μm), and samples were measured by LC-MS/MS.

### Food preparation and food analysis

Ripe strawberries were purchased at local supermarkets in Freising, Germany. Strawberries were thoroughly blended using a commercial hand mixer. The blended fruits were analyzed for folates and used as a test food in the human study. Strawberries were analyzed for folates according to a previously described validated SIDA [28].

### Instrumental conditions

#### HPLC-DAD

Determination of the unlabeled 5-CH_3_-H_4_folate was performed on a Shimadzu HPLC/DAD system (Shimadzu, Kyoto, Japan) using a reversed phase column (C18 EC, 250 × 3 mm, 5 μm, 100 Å, precolumn: C18, 8 × 3 mm, Machery-Nagel, Düren, Germany) for separation. The mobile phase consisted of a binary mixture of 0.1% aqueous acetic acid (A) and methanol (B) at a flow rate of 0.4 mL/min. The gradient solution started at 10% B for 7 min, followed by raising the concentration of B linearly to 50% during the next 14 min. Next, the mobile phase increased to 100% B within 2 min and maintained at 100% B for a further 1 min. The mobile phase was returned to an initial concentration of 10% within 2 min before equilibrating the column for 9 min. The injection volume was 10 μL, and the analysis was performed at room temperature.

#### LC-MS/MS

The plasma samples were chromatographed on a Shimadzu Nexera X2 UHPLC system (Shimadzu, Kyoto, Japan) with a Raptor ARC-18 column (2.7 μm, 100 × 2.1 mm, Restek, Bad Homburg, Germany) and a Raptor ARC-18 precolumn (2.7 μm, 5 × 2.1 mm, Restek, Bad Homburg, Germany) as a stationary phase that was kept in the column oven at 30°C. The binary gradient system consisted of 0.1% aqueous formic acid (A) and acetonitrile (B) with 0.1% formic acid at a flow rate of 0.4 mL/min. The gradient started at 3% B and was raised linearly to 10% B within 2.5 min and maintained at 10% B for 2.5 min. Subsequently, the mobile phase was increased to 15% B during the next 5 min and to 50% B during the further 1 min. The mobile phase was then held at 50% for 1 min before equilibrating the column for 4 min with the initial concentration of 3% B. The injection volume was 10 μL.

The LC was interfaced with a triple quadrupole ion trap mass spectrometer (LC MS-8050, Shimadzu, Kyoto, Japan), operated in the positive ESI mode. The ion source parameters were set as follows: heat block, dilution line, and interface temperature were set to 400°C, 250°C, and 300°C, respectively. The flow rates of the drying, heating, and nebulizing gases were set to 10, 10, and 3 L/min, respectively. Collision-induced dissociation gas was applied at 270 kPa, and interface voltage was applied at 4 kV. MS parameters were optimized by injection of an unlabeled 5-CH_3_-H_4_folate solution (1 μg/mL). The optimized parameters were assumed for the respective isotopological internal standard. The mass spectrometer was operated in the multiple reaction monitoring (MRM) mode for MS/MS measurements under conditions detailed in Table 1. The first MRM transition represents the ion for quantification, and the second MRM transition is used to verify the qualification. The [^13^C_5_]-labeled glutamyl residue split off during fragmentation, whereas the analyte fragments and the respective internal standards were the same. The relative abundance of the second transition to the first transition in the matrix in comparison with the pure standard solution verified the analyte as well as the peak purity. To remove the first eluting salt particles and to avoid salt particles from entering the mass spectrometer, a waste valve diverted the column effluent to the mass spectrometer only between 2.1 to 7 min. Data acquisition was performed with the LabSolutions software 5.8 (Shimadzu, Kyoto, Japan).

**Table 1:** MRM scan parameters for 5-CH_3_-H_4_folate and the respective internal standard [^13^C_5_]-5-CH_3_-H_4_folate.

Compound	Precursor [m/z]	Product [m/z]	Dwell Time [msec]	Q1 Pre Bias [V]	CE [V]	Q3 Pre Bias [V]
**5-CH**_**3**_**-H**_**4**_**Folate**	460.2	313.20	50.0	-13.0	-20.0	-17.0
		180.15	50.0	-13.0	-37.0	-14.0
		194.25	50.0	-23.0	-33.0	-22.0
**[**^**13**^**C**_**5**_**]-5-CH**_**3**_**-H**_**4**_**Folate**	465.3	313.2	50.0	-13.0	-20.0	-17.0
		180.15	50.0	-13.0	-37.0	-14.0
		194.25	50.0	-23.0	-33.0	-22.0

### Method validation

#### Calibration and quantitation

For the response curve, constant amounts of internal standard (S) were mixed with varying amounts of analyte (A) in the molar ratios $n\left(A\right)/n\left(S\right)$ between 0.03 and 14.9 for preparing 12–13 calibration points. Polynomial regression was used by combining the molar ratios $n\left(A\right)/n\left(S\right)$ with the peak areas $A\left(A\right)/A\left(S\right)$ from the LC-MS/MS measurements.

#### Limits of detection and quantification

Limits of detection and quantification (LOD, LOQ) were determined according to Vogelgesang and Hädrich [29]. For determining the LOD and LOQ, 60 μL of sample volume was used. A mixture of 0.1% egg white, 0.1% sunflower oil, 0.9% sodium chloride, and 98.9% water was used as a blank matrix. The blank matrix was analyzed to confirm the absence of folates. Subsequently, 60 μL of matrix was spiked with the unlabeled 5-CH_3_-H_4_folate at four different concentrations, with the lowest concentration being slightly above the estimated LOD and the highest concentration being 10-fold higher (6.16 nmol/L; 12.3 nmol/L; 15.4 nmol/L; 67.0 nmol/L). Each concentration level was determined in triplicate.

The calculated data obtained from SIDAs and the known spiked amounts were correlated. According to a previously published method [29], a subsequent regression calculation was used for the calibration line and the confidence interval was used to calculate the LOD and LOQ.

#### Precision

Original plasma samples were used for precision measurements. The precision measurements were performed using 30 and 60 μL sample volume. Inter-day precisions, inter-injection assays, and intra-day precisions were determined. For determining the inter-day precision, plasma samples were analyzed in triplicate, and experiments were repeated thrice within 2 weeks. The relative standard deviation of the three independent extractions represents the inter-day precision. Inter-injection assays were determined by injecting one sample 15 times in a row in the LC-MS/MS. The inter-injection assay represents the precision of the instrument. For determining the intra-day precisions, identical plasma sample was extracted in triplicate. The relative standard deviation was calculated in %.

#### Recovery of SIDAs

Recoveries were determined using 60 μL sample volume. Blank matrices (60 μL) were spiked in triplicate using unlabeled 5-CH_3_-H_4_folate in three different concentrations (15.1 nmol/L; 60.4 nmol/L; 106 nmol/L). The samples were analyzed as previously described, and the recoveries were calculated as the ratio of the detected and the spiked contents.

### Biokinetic calculations

For determining the plasma concentration curve, 5-CH_3_-H_4_folate levels were measured. Plasma concentration curve of placebo test day was subtracted from the plasma concentration curve of the strawberry and supplement test day. The concentrations were subjected to a standard analysis in order to calculate c_max_ (maximum plasma concentration), t_max_ (time of c_max_), and area under the postresorptive plasma concentration-time curve (AUC). The AUC was calculated by linear trapezoidal rule. The relative bioavailability of strawberries was determined in relation to the 5-CH_3_-H_4_folate tablet; therefore, we assumed a 100% absorption of the supplement. All samples were determined in technical triplicates and a three-fold injection of each replicate. The values were tested for normal distribution with the test of David and were tested for outliers by the test of Dixon. A significance test was carried out by using the T-test after applying the F-test for determining homoscedasticity and heteroscedasticity. The level of statistical significance was set to p < 0.05.

## Results

**Fig 2:**
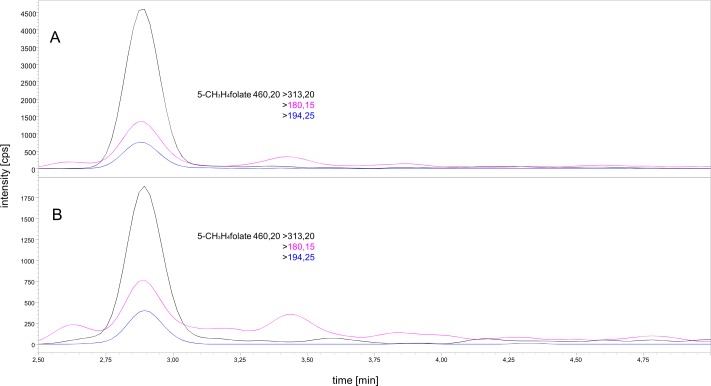
Comparison of extraction chromatograms of 60 μL (A) and 30 μL (B) of plasma; 5-CH_3_-H_4_folate m/z 460.20→313.20 (black), 460.20 → 180.15 (pink), and 460.20 → 194.25 (blue).

**Fig 3:**
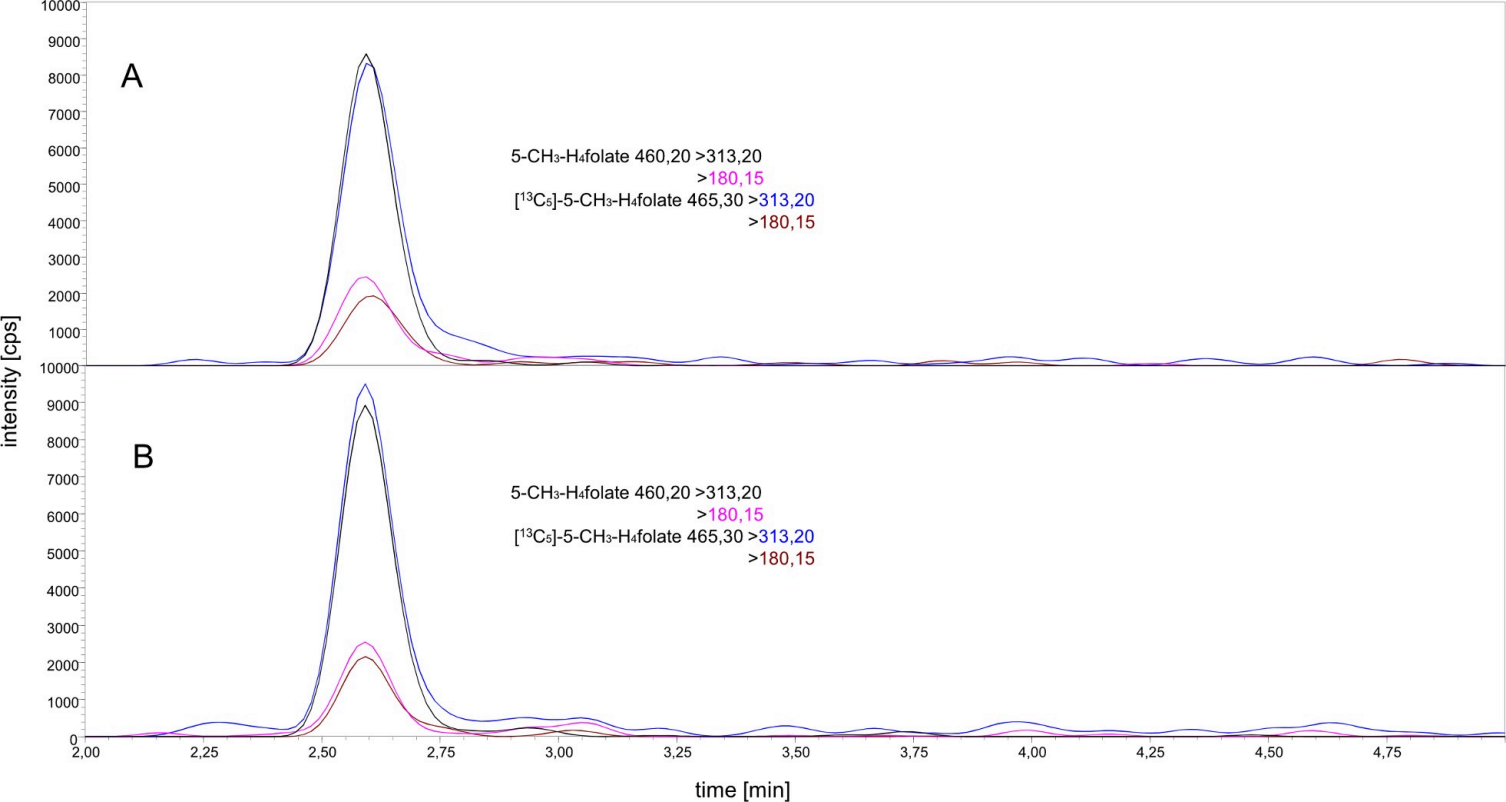
Comparison of the matrix effect of K3 EDTA and lithium-heparin. Extraction of 60 μL plasma drawn with a K3 EDTA coated tube (A) and lithium-heparin coated tube (B); 5-CH_3_-H_4_folate m/z 460.20 → 313.20 (black), 460.20 → 180.15 (pink), and [^13^C_5_]-5-CH_3_-H_4_folate m/z 465.30 → 313.20 (blue), 460.30 → 180.15 (brown).

The method for quantitation of 5-CH_3_-H_4_folate was validated for performing a human study. LC-MS was carried out as previously described [28]. The application of a UHPLC-method enables a high sample throughput. Particularly, for future bioavailability studies, where a sufficient number of blood samples are necessary [30] and an AUC of at least 80% needs to be detected [31], a fast and effective LC-MS method is necessary. For quantitating the metabolized plasma folate form, we used [^13^C_5_]-5-CH_3_-H_4_folate as an internal standard. However, for detecting the vitamers tetrahydrofolate (H_4_folate), 5-formyltetrahydrofolate (5-CHO-H_4_folate), 10-formyl-folic acid (10-CHO-PteGlu), and pteroylmonoglutamate (PteGlu), we added mass transfers to our method. Sample extraction was carried out as described by Mönch et al. [20] with slight modifications. In the first analytical step, we tried to reduce the previously established sample volume of 400 μL used by Mönch et al. [20]. The chromatogram displayed in Fig 2 indicates the extraction of folates from 60 and 30 μL sample volumes, revealing an intensity considered to be satisfactory for analyte quantitation. The first mass transfer (465.30 > 313.20, black color) was used for quantitation. We calculated an estimated LOD to be three-fold above the S/N of an intensity of 600 cps. The intensities of the 60 and 30 μL samples were 4500 and 1750 cps, respectively, which was sufficient for an accurate quantitation. To investigate sensitivity of the assay, we extracted 60 and 30 μL of sample volumes of an identical plasma sample from venous blood in triplicate. The 5-CH_3_-H_4_folate content in case of the 60 and 30 μL of plasma samples were 21.3 and 25.3 nmol/L, respectively. Thus, different sample volumes did not lead to significantly (p > 0.05) different results. Besides, lowering the sample volume to a minimum was an important aim, which was needed for a reliable analysis of finger prick blood. The minimum limit of sample volume is primarily limited by the sensitivity of the mass spectrometer. It is also dependent on the blood volume received from a single finger prick, which varies on an individual basis. Further, we tested different microvettes with either cylindrical or conical shape of the inner tube, with K_3_EDTA and lithium-heparin for anti-clotting, and with or without capillary blood collection. A one-time finger prick puncture usually resulted in around three to four blood drops, which corresponded to 50 μL–60 μL of whole blood. Finger prick blood was directly collected in microvettes and centrifuged to separate plasma. A maximum volume of 30 μL plasma was obtained out of a one-time puncture in all tests. In order to carefully separate the plasma from the red blood cells using a pipette, an inner tube with a conical shape was necessary. Since microvettes with such a shape were only available coated with lithium-heparin, we tested the matrix effects of K_3_EDTA and lithium-heparin on the mass spectrometer. Since no differences in intensities of 5-CH_3_-H_4_folate were observed (Fig 3), we decided to use 100 μL microvettes with lithium-heparin and with a conical inner shape of the tube. Regarding the validity of finger prick blood compared to venous blood, Kopp et al. [32] compared the validity of finger prick blood in comparison with venous blood and did not find significant differences. Moreover, for an application test of the finger prick method, please refer to supporting information (S1 Text).

### Validation

The validation data are summarized in Table 2.

**Table 2:** Validation of the stable isotope dilution assay.

Analyte	LOD	LOQ	Precision (n = 3) [RSD%]			Recovery [%]		
	[nmol/L]	[nmol/L]	Inter-injection	Intra-day	Inter-day	Spiking level 1	Spiking level 2	Spiking level 3
5-CH_3_-H_4_folate	1.51	4.51	3.70 (60 μL	3.35 (60 μL)	3.66 (60 μL)	102 (15.1 nmol/L)	101 (60.4 nmol/L)	99.3 (106 nmol/L
			7.42 (30 μL)	4.51 (30 μL)	5.02 (30 μL)			

The LOD, LOQ, and recoveries were determined in a matrix of egg white, sunflower oil, sodium chloride, and water in a sample volume of 60 μL. Precision was determined in blood plasma related to a sample volume of 60 and 30 μL.

### Calibration of SIDA

The response function for 5-CH_3_-H_4_folate was obtained using polynomial regression after testing linearity according to Mandel. The calibration curve was polynomial within the molar ratios $n\left(A\right)/n\left(S\right)$ between 0.03 and 10.1 $\left(y=-0.0034x^{2}+0.7991x-0.0015,R^{2}=0.9999\right)$ [28]. The response equation was calculated as follows after plotting the molar concentration ratios of the analyte and internal standard on the y-axis and peak area ratios of the analyte and internal standard on the x-axis (1):

$$n\left(A\right)/n\left(S\right)=m_{1}\times {\left[\frac{A\left(A\right)}{A\left(S\right)}\right]}^{2}+m_{2}\times A\left(A\right)/A\left(S\right)+b$$ (1)

#### LOD and LOQ

Since 60 μL of blank matrix was expected to show higher matrix effects and therefore likely to exhibit a higher LOD and LOQ than a 30 μL blank matrix, we decided to use 60 μL of sampling volume for determination of the LOD and LOQ of both volumes (Table 2). The LOD and LOQ for 5-CH_3_-H_4_folate were 1.51 and 4.51 nmol/L, respectively.

#### Precision

The inter-day precision for 60 and 30 μL sample volume was 3.66% and 5.02%, respectively. The intra-assay precision was 3.35% and 4.51% for 60 and 30 μL, respectively, whereas the inter-injection assay precision was 3.70% and 7.42% for 60 and 30 μL, respectively (Table 2).

#### Recoveries of SIDA

The recoveries for the spiking concentration of 15.1, 60.4, and 106 nmol/L were 102%, 101%, and 99.3%, respectively (Table 2). According to Vogelgesang and Hädrich [29], recoveries should ideally lie between 70% and 120%; our results deviate by a maximum of 2% from the calculated concentrations. This can be explained by the reduced number of purification steps and very few error sources during the extraction procedure.

### Performance of a human short-term study

The validated method using 60 μL venous blood was applied to a human pilot study. The test person was required to perform three independent test days designed as follows. To confirm a constant plasma concentration over the study day, a placebo test day was necessary. For the strawberry test day, the test person was required to consume 600 g of strawberries. For the supplement test day, the test dose consisted of a commercial 5-CH_3_-H_4_folate supplement (400 μg 5-CH_3_-H_4_folate). In order to simulate possible matrix effects, the supplement was parallely administered with a water-pectin-sugar mixture. A previously published method was applied for determining the exact dose of folate from strawberries [28].

**Fig 4:**
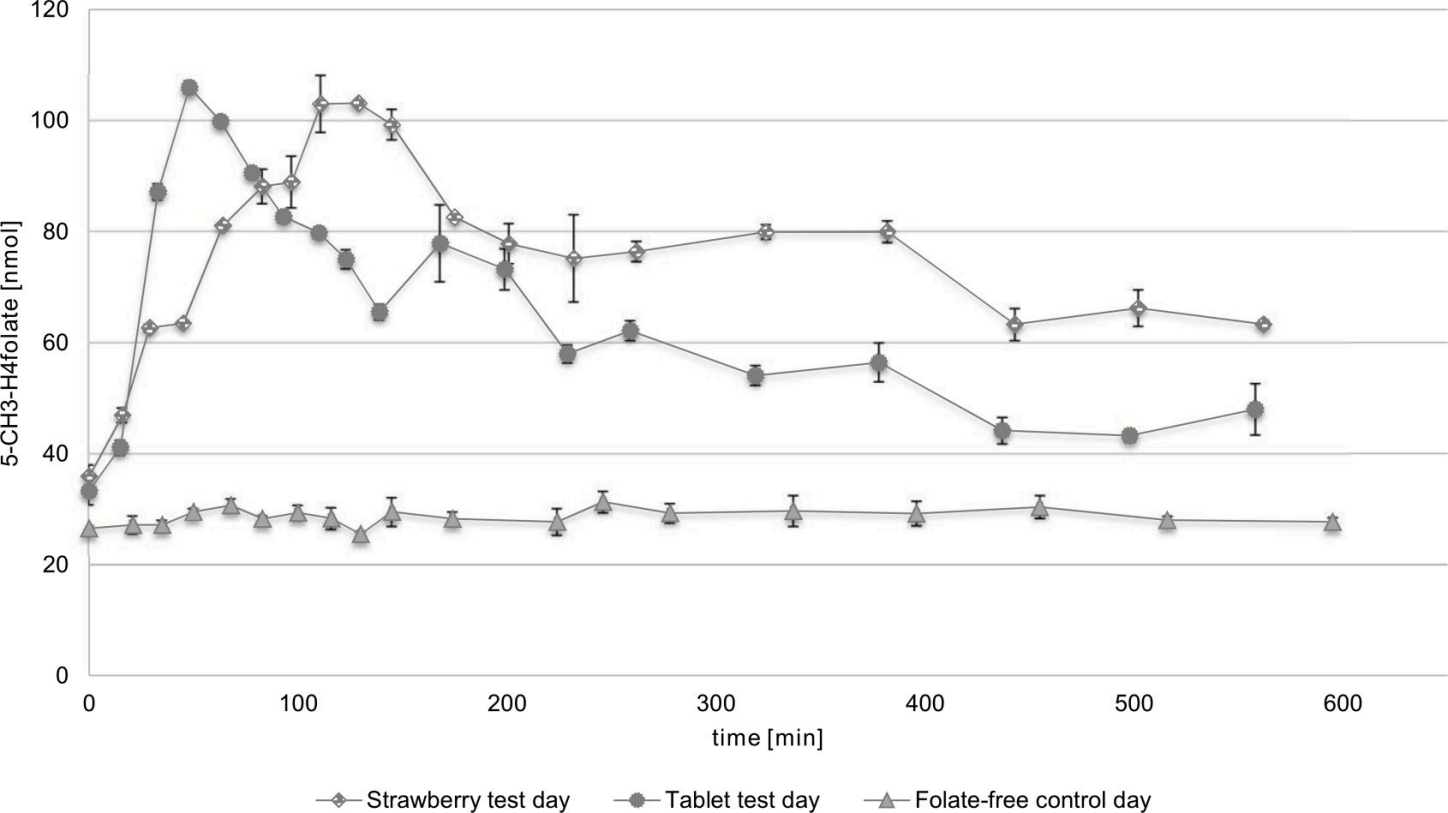
Postresorptive plasma concentration on the folate-free control day, strawberry test day, and tablet test day (subject 1). Standard deviation [nmol], calculated from a technical triplicate.

To determine the postresorptive plasma concentration curve, blood was drawn once before consumption and then up to 10 h after consumption of the test food. The mean plasma concentrations of 5-CH_3_-H_4_folate are shown in Fig 4. Table 3 summarizes the kinetic parameters of 5-CH_3_-H_4_folate. Besides 5-CH_3_-H_4_folate, all samples were tested for PteGlu, H_4_folate, and 5-CHO-H_4_folate, where no further vitamers were detected. The folate-free control day showed a constant plasma concentration over time, whereas the postresorptive plasma concentration of the strawberry and tablet test day revealed a considerable increase after the consumption of the respective food. The 5-CH_3_-H_4_folate concentration of the folate-free control day varied between 25.5 and 31.3 nmol/L.

**Table 3:** Summary of biokinetic parameters of the folate-free test day, strawberry test day, and the tablet test day (subject 1).

study day	study time [h]	absolute 5-CH_3_-H_4_folate [μg]	predose value	c_min_ [nmol/L]	c_max1_ [nmol/L]	c_max2_ [nmol/L]	t_max1_ [h]	t_max2_ [h]	AUC [nmol/Lxh]	rel. BV [%]
**Folate-free control day**	9.33	-	26.5	25.5	31.3	-	-	-	286	
**Strawberry test day**	9.37	615	37.4	37.4	103	80.0	2.15	6.37	707	96.2
**Tablet test day**	9.30	400	32.9	32.9	106	77.9	0.80	2.80	571	

The plasma concentrations on the strawberry test day before food consumption was 35.9 nmol/L. After administration of strawberries, the 5-CH_3_-H_4_folate concentration increased significantly (p < 0.05) to 103 nmol/L (c_max1_) after 2.15 h (t_max1_), which complies with an increase of 187%. During the subsequent 1.72 h, the plasma folate concentrations showed a decline of 73% and reached c_max2_ of 80.0 nmol/L after 6.37 h (t_max2_). Until the end of the study period, the folate concentration decreased slightly to 63.3 nmol/L. Further, the plasma concentration at the end of the study was significantly (p < 0.05) higher (76.3%) compared with the value obtained before administration of the respective test food.

The supplement test day showed a similar trend as that of the postresorptive plasma curve compared with the strawberry test day. After administration of a 5-CH_3_-H_4_folate supplement, the plasma concentration increased significantly (p < 0.05) from 33.3 to 106 nmol/L (c_max1_) after 0.8 h (t_max1_), which equals a 219 percentage increase. The plasma concentration decreased in time to 38.2% and reached c_max2_ with 77.9 nmol/L after a total of 6.91 h (t_max2_). Until the end of the study day, the 5-CH_3_-H_4_folate concentration decreased continuously and the concentration by the end of the study was 48.0 nmol/L.

To compare the values on the strawberry and the supplement test day, the relative bioavailability of strawberries compared with a 5-CH_3_-H_4_folate tablet was calculated using the AUC method. The total observed folate content of strawberries, calculated as PteGlu and as 5-CH_3_-H_4_folate, was 95.9 ± 3.24 μg/100 g and 99.8 ± 3.24 μg/100 g, respectively. With a share of 94.8%, the major folate vitamin was 5-CH_3_-H_4_folate. Minor vitamers were 5-CHO-H_4_folate and H_4_folate with 4.27% and 0.96%, respectively. The absolute administration of folates was 615 μg 5-CH_3_-H_4_folate on the strawberry test day and 400 μg 5-CH_3_-H_4_folate on the tablet test day. The absolute dose of folate was taken into account when calculating its relative bioavailability; a relative bioavailability of 96.2% of strawberries to the supplement was calculated.

The three treatments were applied to a further test person; please refer to the supporting information (S1 Table and S1 Fig). However, the determination of the 5-CH_3_-H_4_folate concentration was performed using the method of Kopp and Rychlik [32]. The treatments were performed in the same way as described before. For the second test person, we calculated a relative strawberry folate bioavailability of 73.3%. These results exhibit a similar trend and support the findings presented above.

## Discussion

A method capable for monitoring the folate status using finger prick as well as for performing bioavailability studies was validated. The LOD value was slightly higher than the previously reported value of 0.8 nmol/L [20] by using a 400 μL sample volume. Usually, the LOD and LOQ are calculated in relation to the sample weight. However, the calculated absolute LOD was 90.6 mmol in contrast to 320 mmol as previously reported [20] and thus the sensitivity was increased about 3.5 fold. Our results were consistent with the plasma folate analysis reported by Kopp and Rychlik [32], where an LOD and LOQ of 1.5 and 4.4 nmol/L, respectively, was obtained for dried plasma spots and an LOD and LOQ of 2.2 and 6.3 nmol/L, respectively, was obtained for dried blood spots for plasma. Since the latter authors used 50 μL of sample volume, the results can easily be compared. The precision values are similar compared with previous reports [20, 33]. Furthermore, we received equally good recoveries in comparison with Mönch et al. [20]. Compared with Kopp and Rychlik [32], who received recoveries from 80% to 116% for dried plasma spots and dried blood spots for plasma, we obtained better recoveries between 99.3% and 102%. The deviation in recoveries might partly be attributed to the fact that in contrast to our method, other methods needed an additional heating and ultrasonication step.

The validated method was applied to an *in vivo* pilot study for evaluating the bioavailability of folates from strawberries. The method allowed a precise determination of a postresorptive plasma concentration curve. The biological triplicates showed low relative standard deviation between 0.04% and 10.5% (standard deviation between 0.03 nmol/L and 7.85 nmol/L). Accordingly, significant differences (p = 0.05) in a very low concentration range can be calculated.

The folate storage pools in volunteers were saturated for standardization and to minimize the hepatic first-pass uptake. A standardized low-folate diet plan minimized the physiological differences and ensured identical dietary conditions for each treatment. The diet plan included regular consumption of a low-folate diet in order to maintain folate homeostasis and to prevent variability caused by fasting and disruption of the enterohepatic circulation [34, 35].

The plasma concentration during the folate-free control day was constant during the day. To ensure that the diet plan did not result in an increase of plasma folate concentration, the performance of a folate-free control day was crucial for determination of the relative bioavailability. However, recent studies have often used only the first blood sample drawn prior to consumption of the test food as a control [36].

In an earlier study, Castenmiller et al. [37] described a slightly higher bioavailability of pooled, minced, and liquefied spinach compared with whole-leaf spinach. Although we minimized the matrix effect of strawberries, an incomplete release from the matrix cannot be excluded. However, a relative bioavailability of 96.2% can be considered as an almost complete release from the matrix. Since both study days were performed in an identical way, the incomplete bioavailability of 73.3% cannot be attributed to an incomplete release from the matrix.

In case of both treatments, the time needed to attain the maximum concentration was different between the strawberry and the supplement test day. T_max1_ and t_max2_ of the supplement test day were achieved earlier compared with the strawberry test day. This indicated that the deconjugation of the polyglutamates of the naturally occurring folates to the absorbable monoglutamates is a time limiting step. Witthöft et al. [38] observed similar results and a later t_max1_ after administration of strawberries and broccoli compared with a 5-CH_3_-H_4_folate solution. The earliest t_max1_ was observed after intramuscular injection, where no intestinal absorption was necessary. Furthermore, our study showed that the plasma concentration on the supplement test day increased fast but also decreased faster until the end of the study day. In contrast, the plasma concentration on the strawberry test day was higher at the end of the study day. This indicates that the uptake of natural folates, more specifically the cleavage of polyglutamates as well as encapsulation of folates of the matrix, takes longer. However, the net effect until the end of the intervention is similar to that of synthetic monoglutamates. Furthermore, the relative bioavailability of 96.2% (subject 1) and 73.3% (subject 2) is very high compared with previous studies. For instance, the relative bioavailability of low-fat camembert using a PteGlu solution as a reference was reported to be 65–71% [39] and that of spinach using a 5-CH_3_-H_4_folate solution as a reference was reported to be between 47% and 53% [38]. This result may be attributed to vitamer distribution in strawberries; the main vitamer in strawberries was analyzed to be 5-CH_3_-H_4_folate, which is the metabolized form in plasma. Mönch et al. [40] also found a higher bioavailability of spinach compared with camembert and wheat germs, the former containing a higher percentage of 5-CH_3_-H_4_folate. The remarkable points of the postresorptive plasma concentration curve were not just the increase of plasma folate and c_max1_ after consumption of the respective test food but also c_max2_, which appeared after 6.37 h (t_max2_) on the strawberry test day and after 6.91 h (t_max2_) on the supplement test day. To the best of our knowledge, this is the first report about a second increase of folate plasma concentration (c_max2_) after the consumption of a test food. Steinberg et al. [34] already proved using a rat model that 5-CH_3_-H_4_folate is quantitatively excreted into the bile after hepatic first pass, reabsorbed and distributed into tissues. A bile drainage led to a decrease in serum folate. Pratt and Cooper [41] reported remarkable biliary levels of labeled folates in humans after administration. We assume that the c_max2_ of plasma folate is a result of resorption. More specifically, we believe that the major vitamer of strawberries, 5-CH_3_-H_4_folate, is transported into the bile and then reabsorbed for distribution to tissues and the liver. Our results support the assumption made in earlier works that the enterohepatic circulation plays a key role in folate homeostasis [42].

## Conclusions

The validated SIDA presented here is a reliable and an accurate method for analysis of plasma folate levels. The reduction of sample volume enabled a reduction of usage of the cost-intensive internal standards. The method exhibited very high sensitivity and precision compared with previously reported SIDAs. Additionally, the compatible application of finger prick blood and venous blood showed to be very beneficial. Using finger prick, the method proved to be applicable in monitoring the individual folate status. Due to the use of finger prick blood, no medical surveillance is necessary for monitoring the folate status. More specifically, the method is a step forward towards simplify the implication of surveys about folate status in countries facing challenges in logistics and lack of medical surveillance. The use of finger prick blood allows an easier data collection about folate status in such populations. Moreover, the use of a very small (i.e., 60 μL) sample volume enables an accurate and precise detection of a postresorptive plasma curve after administration of a test food and enables the detection of even slight differences in 5-CH_3_-H_4_folate concentrations. Furthermore, due to the implementation of UHPLC, a high-throughput of samples is possible. This method proved suitable for determination of folate bioavailability from various foods.

Primarily, the present work confirmed once again that strawberries are a rich natural source of folates. This study showed on a pilot scale that folates from strawberries are highly bioavailable and absorbable for the human body. However, the deconjugation process and the encapsulation of matrix may apparently be a limiting factor in the absorption process of strawberry folates. Furthermore, the folate content of strawberries itself can be influenced by factors such as cultivars, maturity, climate, and duration of storage [28, 43, 44]. Due to the important role of folates in human health, accurate dietary recommendations are crucial for an adequate folate intake. More specifically, in countries without mandatory folate fortification, knowledge about good natural folate sources and their availability for absorption can substantially support human folate status and human health.

With respect to the current pilot study, strawberries contribute to reducing folate deficiency. However, since the different subjects may show substantial variation in response, further investigations and human studies with a higher number of enrolled subjects should be planned to enable an improved statistical and biokinetic interpretation of the data. Although our results indicate that strawberries are an important source of folates, long-term studies are required to establish the extent to which folate from strawberries may reduce plasma homocysteine and evaluate long-term changes in storage folates.

In summary, human studies performed using accurate methods for the quantitation of the food folate content as previously presented [28], and blood folate content as presented here offers prospects for improved recommendations for folate intake, especially in women of childbearing age.

## Supporting information

S1 Text Application of the finger prick method. (DOCX)

S1 Table Summary of biokinetic parameters observed on the folate-free test day, strawberry test day, and the tablet test day (subject 2). Extractions were performed as described previously [31].
(DOCX)

S1 Fig Postresorptive plasma concentration on the folate-free control day, strawberry test day, and the tablet test day (subject 2). Standard deviation in [nmol], calculated from a technical triplicate. Extractions were performed as described previously [31].
(TIF)
