# Supplementary material for: Quantitation of 5-methyltetraydrofolic acid in plasma for determination of folate status and clinical studies by stable isotope dilution assays
Source: PLoS One. 2019 Feb 21;14(2):e0212255. doi: 10.1371/journal.pone.0212255 (PMC6383923; doi:10.1371/journal.pone.0212255)
Supplement: S1 Text — (DOCX) [file pone.0212255.s001.docx]

**Supporting information**

S1 Text: Application of the finger prick method

A small application test using the finger prick method was performed with two test persons. The results of test person one was 22.2±1,58 nmol/L and test person two was 20.5±3.63 nmol/L. The results are in the normal range of an expected plasma folate concentration.
