# Supplementary material for: Quantitation of 5-methyltetraydrofolic acid in plasma for determination of folate status and clinical studies by stable isotope dilution assays
Source: PLoS One. 2019 Feb 21;14(2):e0212255. doi: 10.1371/journal.pone.0212255 (PMC6383923; doi:10.1371/journal.pone.0212255)
Supplement: S1 Table — Extractions were performed as described previously [31]. (DOCX) [file pone.0212255.s002.docx]

**Supporting information**

S1 Table: Summary of biokinetic parameters observed on the folate-free test day, strawberry test day, and the tablet test day (subject 2). Extractions were performed as described previously [31].

| **study day** | **study time [h]** |  | **absolute 5-CH_3_-H_4_folate [µg]** | **predose value** | **c_min_ [nmol/L]** | **c_max1_ [nmol/L]** | **c_max2_ [nmol/L]** | **t_max1_ [h]** | **t_max2_ [h]** | **AUC [nmol/Lxh]** | **rel. BV [%]** |
| --- | --- | --- | --- | --- | --- | --- | --- | --- | --- | --- | --- |
| **folate-free control day** | 9.12 |  | - | 20.3 | 20.3 | 36.3 | - | - | - | 253 |  |
| **strawberry test day** | 9.38 |  | 671 | 50.4 | 50.4 | 101 | 93.7 | 1.37 | 3.32 | 767 | 73.3 |
| **tablet test day** | 8.98 |  | 400 | 57.6 | 57.6 | 86.1 | 78.8 | 0.93 | 2.25 | 671 |  |
