# Supplementary figures and images for: Quantitation of 5-methyltetraydrofolic acid in plasma for determination of folate status and clinical studies by stable isotope dilution assays
Source: PLoS One. 2019 Feb 21;14(2):e0212255. doi: 10.1371/journal.pone.0212255 (PMC6383923; doi:10.1371/journal.pone.0212255)

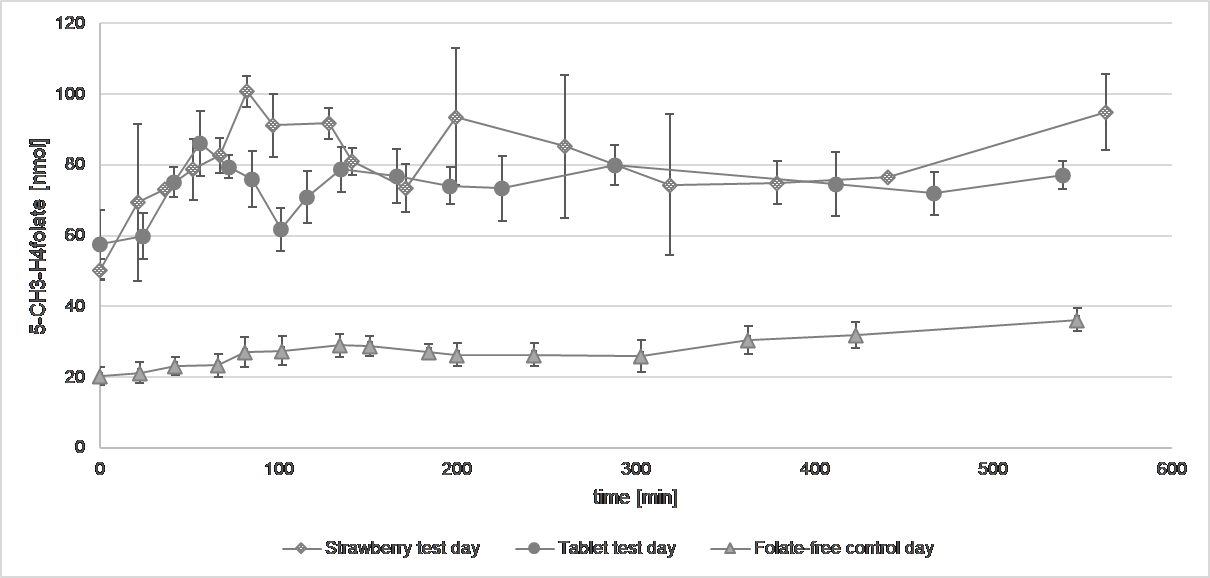

Supplement: S1 Fig — Standard deviation in [nmol], calculated from a technical triplicate. Extractions were performed as described previously [31]. (TIF) [file pone.0212255.s003.tif]
